# Editorial: Redox Regulation in Skeletal Muscle Aging and Exercise

**DOI:** 10.3389/fphys.2016.00005

**Published:** 2016-01-22

**Authors:** Brian McDonagh

**Affiliations:** MRC-Arthritis Research UK Centre for Integrated Research into Musculoskeletal Aging, Institute of Ageing and Chronic Disease, University of LiverpoolLiverpool, UK

**Keywords:** skeletal muscle aging, redox signaling, exercise

Changes in population demographics have seen an increase in human lifespan coupled with an increase in many age associated disorders that determine quality of life including, sarcopenia, and frailty. Closing the gap between life expectancy and healthy aging has now become a research priority in many countries. Skeletal muscle comprises up to 40% of body mass and the inhibition or delay of the progressive loss in muscle mass with age would help maintain mobility, strength and independence. Regular exercise is one of the few interventions known to help maintain both muscle mass and strength with age. The metabolic and structural changes induced by exercise ultimately affect the contractile properties of the muscle fiber. The role of endogenously generated reactive oxygen and reactive nitrogen species (ROS/RNS) as intracellular signaling molecules have been identified as playing a crucial role in the correct adaptation and response to exercise in skeletal muscle. The species, concentration and cellular location of endogenously generated ROS/RNS can directly and indirectly affect the activity of redox regulated proteins and transcription factors with a multitude of downstream effects. There are a number of potential sites for ROS/RNS generation including electron leakage from the electron transport chain, but more recent studies have identified endogenous cytoplasmic sources as a major contributor to ROS/RNS generation within muscle during contractions (Sakellariou et al., 2013; Pal et al., 2014; Pearson et al., 2014). An attractive mechanism for the redox regulation of numerous cellular processes is a redox signaling relay from cytoplasmic generation to a mitochondrial response, through the sequential modification of specific residues in regulatory proteins located in close proximity to the sites of endogenously generated ROS/RNS (McDonagh et al., 2014). Understanding the mechanisms of redox regulation in skeletal muscle and the changes induced during aging or disease are essential to maximize the beneficial effects of exercise. This special topic is a collection of original research and review articles that aims to bring together recent advances in our understanding of redox regulation in skeletal muscle during aging and exercise.

Elevated levels of Sphingomyelinase (SMase) have been detected during aging and in a variety of diseases, SMase induced sphingolipid metabolism results in a decrease in force production, increased fatigue and muscle atrophy (Ferreira et al., 2012). In the article by Lehr and colleagues they utilized redox sensitive green fluorescent probes (roGFP) targeted to Nox2 or the mitochondria allowing the identification of the site specific ROS generation as a result of SMase stimulation in single muscle FDB fibers (Loehr et al.). Results identify Nox2 as the major enzyme responsible for the increase in ROS, which was confirmed by a lack of increased ROS generation in Nox2^−∕*y*^ animals and Nox2 deficiency offers partial protection against SMase induced fatigue (Loehr et al.). Claflin and co-workers have contributed a novel method to assess mitochondrial function in skeletal muscle by fluorescently monitoring NADH/NAD^+^ balance as a reflection of the global mitochondrial redox state (Claflin et al.). They performed lengthening procedures on isolated lumbrical muscles from adult and old mice deficient in CuZnSOD, allowing force measurements and fluorescent monitoring of NADH concentrations (Claflin et al.). A brief period of intense contractions resulted in a large, reproducible fluorescence oscillation of NADH that ultimately returned to pre-contraction levels. Differences in the duration and magnitude of oscillations from adult and old mice, was attributed to differences in the ROS buffering capacity of old mice (Claflin et al.). Continuing with mitochondrial redox signaling cascades as a result of skeletal muscle contractions and exercise, Brandauer and colleagues investigated the requirement of AMPK for the exercise induced increase in the mitochondrial deacetylase SIRT3 and its target mitochondrial proteins (Brandauer et al.). Activation of AMPK by exercise or pharmacologically, can increase PGC-1α phosphorylation with subsequent increases in SIRT3 abundance and activation of its downstream targets (Brandauer et al.).

Skeletal muscle T-tubules are essential for excitation-contraction coupling and form triads with adjacent terminal cisternae of the sarcoplasmic reticulum regulating Ca^2+^ release and sequestration. Triads are unusual in that they contain high levels of cholesterol and sphingolipids, the group of Hidalgo has investigated the effects of cholesterol removal and age, on excitation-contraction (E-C) coupling and the protein content of triads in single muscle fibers (Barrientos et al.). A reduction in cholesterol impaired E-C coupling as a result of a modification of the interactions of the cholesterol associated caveolin-3, with Cav1.1 and the ryanodine receptor, interestingly the levels of the cholesterol binding caveolin-3 protein also decreased with age (Barrientos et al.).

Two contributions from the O'Halloran group examine the redox remodeling effects of chronic intermittent hypoxia (CIH) and chronic sustained hypoxia (CH) in sternohyoid muscle (Lewis et al.;Williams et al.). In the article by Williams et al, the authors examined the effects of CIH on rat sternohyoid force and power with complementary analysis of a number of redox parameters and enzymatic activities (Williams et al.). The authors conclude that CIH up-regulates NOX expression with concomitant modest oxidative stress, while constitutive NOX activity decreased sternohyoid muscle force and power (Williams et al.). The sternohyoid muscles of mice exposed to CH, with and without antioxidants, were analyzed by 2D redox proteomics and complimented with force measurements and enzyme activities (Lewis et al.). Alterations in redox and expression changes occurred in metabolic proteins with a functional deficit in sternohyoid function that was prevented by antioxidant supplementation (Lewis et al.).

The special topic also contains a number of selective reviews on redox regulation in skeletal muscle, including a contribution from Edward Debold that discusses potential mechanisms underlying muscle fatigue by ROS/RNS. The author examines studies where pre-treatment with ROS scavengers significantly attenuated the development of fatigue and presents evidence that ROS/RNS mediated fatigue involves modifications of muscle regulatory proteins that results in a decrease in Ca^2+^ sensitivity of these proteins (Debold). In the contribution by Michael P. Wiggs, the author outlines how exercise training or preconditioning can help prevent muscle atrophy due to inactivity (Wiggs). The author presents evidence that exercise induced mild oxidative stress, results in the activation of a number of cellular signaling pathways that can ultimately help protect skeletal muscle from disuse atrophy (Wiggs). Kaltsatou and colleagues present a systematic review on uremic myopathy, they identify studies linking renal failure with progressive muscle weakness and premature fatigue (Kaltsatou et al.). Chronic kidney disease is described as a silent epidemic in many developed countries and this review highlights there is a clear but as yet unexplored link between oxidative stress and uremic myopathy (Kaltsatou et al.).

This special topic highlights important advances in the field for studying redox regulation in skeletal during muscle aging and exercise, including the molecular mechanisms underlying the age related loss of muscle mass and function. I would like to especially thank all the contributors for their insightful and elegant original and review articles. I sincerely hope the Special Topic will help provide colleagues in the musculoskeletal and redox field with a platform that will stimulate ideas and experiments for further research.

## Author contributions

The author confirms being the sole contributor of this work and approved it for publication.

## Funding

BM is supported by the Wellcome Trust ISSF Fund (097826/Z/11/Z).

### Conflict of interest statement

The author declares that the research was conducted in the absence of any commercial or financial relationships that could be construed as a potential conflict of interest.
